# ‘TIME’: A Web Application for Obtaining Insights into Microbial Ecology Using Longitudinal Microbiome Data

**DOI:** 10.3389/fmicb.2018.00036

**Published:** 2018-01-24

**Authors:** Krishanu D. Baksi, Bhusan K. Kuntal, Sharmila S. Mande

**Affiliations:** ^1^Bio-Sciences R&D Division, TCS Research, Tata Consultancy Services Ltd., Pune, India; ^2^Chemical Engineering and Process Development Division, CSIR-National Chemical Laboratory (NCL), Pune, India; ^3^Academy of Scientific and Innovative Research (AcSIR), Ghaziabad, India

**Keywords:** time series, microbiome, community state, visualization, clustering, Granger causality algorithm, web server

## Abstract

Realization of the importance of microbiome studies, coupled with the decreasing sequencing cost, has led to the exponential growth of microbiome data. A number of these microbiome studies have focused on understanding changes in the microbial community over time. Such longitudinal microbiome studies have the potential to offer unique insights pertaining to the microbial social networks as well as their responses to perturbations. In this communication, we introduce a web based framework called ‘TIME’ (Temporal Insights into Microbial Ecology’), developed specifically to obtain meaningful insights from microbiome time series data. The TIME web-server is designed to accept a wide range of popular formats as input with options to preprocess and filter the data. Multiple samples, defined by a series of longitudinal time points along with their metadata information, can be compared in order to interactively visualize the temporal variations. In addition to standard microbiome data analytics, the web server implements popular time series analysis methods like Dynamic time warping, Granger causality and Dickey Fuller test to generate interactive layouts for facilitating easy biological inferences. Apart from this, a new metric for comparing metagenomic time series data has been introduced to effectively visualize the similarities/differences in the trends of the resident microbial groups. Augmenting the visualizations with the stationarity information pertaining to the microbial groups is utilized to predict the microbial competition as well as community structure. Additionally, the ‘causality graph analysis’ module incorporated in TIME allows predicting taxa that might have a higher influence on community structure in different conditions. TIME also allows users to easily identify potential taxonomic markers from a longitudinal microbiome analysis. We illustrate the utility of the web-server features on a few published time series microbiome data and demonstrate the ease with which it can be used to perform complex analysis.

## Introduction

Recent advances in high throughput next generation sequencing technologies and emergence of the field of metagenomics have helped in profiling not only the entire microbial groups in various environment(s), but also enabled cross sectional view of the sample(s) in a longitudinal time scale. While a cross sectional study design aims at comparisons of sample(s) at a single time point, longitudinal studies conduct several observations of the same sample(s) over a regular/irregular time intervals. A cross sectional study can provide insights regarding the differential abundances of the resident microbes across various states which is likely to be an indicator of potentially important biomarkers (Ghosh et al., 2014; Cameron et al., 2017). However, in order to obtain deeper understanding of the inter dependencies as well as periodic patterns and temporal variations in the microbial community, it is essential to perform a longitudinal study (Secrier and Schneider, 2014).

Temporal variation in microbial abundances play a critical role in influencing human health. For example, changes in microbial diversity are known to be associated with flu, seasonal allergies, as well as lifestyle disorders like diabetes and obesity (Hartstra et al., 2015; Riiser, 2015). The decreasing cost per mega-base of sequencing has enabled increased number of such large scale longitudinal metagenomic projects from diverse environments (Caporaso et al., 2011; Parsons et al., 2012; Kato et al., 2015). A number of studies have also concentrated in analyzing the changes in normal human microbiota after a perturbation event like administration of antibiotics (Dethlefsen and Relman, 2011). Unlike the cross sectional studies, the longitudinal microbiome studies have opened a new avenue for understanding the importance of causality analysis and networks based inferences on longitudinal time series microbiome data (Faust et al., 2015). New insights have also been obtained relating to differences in the stability of microbiomes across various environments (Shade et al., 2012). Another study has also elaborated the importance of stationarity analysis and its relation to microbial competition (David et al., 2014).

With the increase in number of microbiome projects, various tools and platforms have been developed for analysis of cross sectional microbiome data (Caporaso et al., 2010; Arndt et al., 2012; Kuntal et al., 2013; McMurdie and Holmes, 2013; Parks et al., 2014; Dhariwal et al., 2017; Kuntal and Mande, 2017). However, most of these tools cannot be utilized for understanding the temporal dynamics of microbial communities obtained from longitudinal studies. The available tools for time series microbiome data analysis are focused for a particular purpose or are implemented as library specific to a software platform (Bucci et al., 2016) which is difficult for biologists inexperienced in programming. While tools like Time-searcher (Hochheiser and Shneiderman, 2004) have options for visualizing any time series data, they have limited functionalities. STEM (Ernst and Bar-Joseph, 2006), TimeClust (Magni et al., 2008) and GATE (MacArthur et al., 2010), developed with a focus on microarray time series data, also cannot be used for time series microbiome data.

In order to obtain meaningful insights from microbiome time series data, we have developed a user friendly GUI web application, called ‘TIME: (Temporal Insights into Microbial Ecology’) publicly available at https://web.rniapps.net/time. ‘TIME’ allows users to upload data and perform analysis by selecting any desired workflow(s). Each workflow is carefully designed to address a biologically relevant question. These analyses include clustering similar taxa based on their temporal behavior, generating causality based inference networks, identification of time point similarities, etc. A new method for clustering time series data is also introduced and implemented in the platform. ‘TIME’ uses powerful visualization techniques coupled with interactive ‘on the fly’ analyses to assist obtaining meaningful inferences from microbiome time series data. Visual data mining and analysis of large time series datasets can be easily performed using this tool, thereby making it convenient for biologists to focus more on the results rather than implementation. ‘TIME’ intends to complement the existing metagenomic analysis tools and incorporate a suite of techniques that are suitable for microbiome time series analysis.

## Results

### The ‘TIME’ Interface and the Workflows

A few time series microbiome studies have sampled data over a reasonably sized longitudinal span from individual(s) or environment(s) (Caporaso et al., 2011). Some of these time series datasets may consist of several short sampling stretches spanning over a long time period (Dethlefsen and Relman, 2011). The ‘TIME’ interface is designed to easily input user data in various formats (described in the “Materials and Methods” section) for visualization and analysis of time series microbiome data. Once the data is uploaded, a summary plot of the richness and diversity of microbial groups at each phylogenetic level is displayed. Following this, a user may proceed analyzing the data step by step selecting a workflow targeted for a specific time series analysis. Various workflows along with their biological implications are discussed below:

#### Workflow-1

##### Identify abundance based variations in taxonomic groups over time

The first and foremost step in any time series analysis pertains to visualization of temporal trends of the constituent entities (for example taxonomic groups in a microbial ecosystem). This workflow can be used to visualize and identify high, medium, and low abundant taxa. In addition, it allows identification of ‘core,’ ‘persistent,’ and ‘transient’ microbial groups which serve as important characteristic constituents of the ecosystem. The core microbiome refers to those taxa which are present across all time-points. On the other hand, the persistent microbiota refers to the ones that are present across extended time points, but not in all. In contrast, the transient group comprises of those sets of taxa which show frequent trends of appearance and disappearance. It should be noted that although the threshold parameters used for defining the ‘core,’ ‘transient,’ and ‘persistent’ have been taken from a previously reported study (Caporaso et al., 2011), they are prone to biases due to sequencing depth.

#### Workflow-2

##### Compare temporal trends between selected taxa

Analysis of time series data often requires trend comparison of a custom set of taxonomic groups. For example, the group may be a set of taxa previously known to show a characteristic behavior. The current workflow allows easy graphical comparison of two or more user selected taxa over the sampled timeline. TIME also allows comparison of trends in microbial abundance using a simple ‘select and plot’ operation, wherein users can choose microbial taxa (at a specified taxonomic level) using a simple auto-complete search or from a dropdown selection. One or more taxa can then be appended to or removed from the existing plot, thereby providing an easy way to study a selected set of microbes. Most of the microbiome datasets consist of sets of highly abundant as well as rare taxa. This poses a major problem while plotting and visualizing multiple taxa together in a single plot, with highly abundant taxa dominating the scale, thereby making it difficult to decipher patterns for the rare groups. In case the selected taxa have different abundance scales, users can utilize the ‘log scaling’ option for comparing their trends. Particular time stretches of interest can also be zoomed for in depth analysis.

#### Workflow-3

##### Identify temporally stable/unstable taxa

One of the important steps in time series analysis pertains to identification of stationary entities corresponding to the ones which have mean, standard deviation and variance constant over time. In microbial time series studies, identification of stationary taxa is especially crucial to detect inter microbial competition (David et al., 2014). As demonstrated in an earlier study (David et al., 2014), the presence of competition among the resident taxa is expected to cause sustained growth of some of them leading to their non-stationary behavior. Since in most cases the microbiota are in stable state, only a few taxonomic groups are expected to be non-stationary. A significant test of non-stationarity hence can be considered as a hint for a restoring force governing bacterial dynamics. However, fluctuations due to diet and environment may also affect stationarity of taxa and hence a cautious interpretation of results is required. Additionally, the similarities in phylogeny of non-stationary taxa may also provide clues pertaining to resource competition as genetically similar taxa are more likely to exhibit resource competition.

#### Workflow-4

##### Identify variations in taxonomic groups between two time ranges

Time series experiments involving perturbation events (like administration of antibiotics) are likely to disrupt the microbial community structure. In such analysis, it might be of interest to identify and visualize the exact temporal effect of the perturbation on the resident microbial groups. This workflow allows identification of taxa which undergo noticeable changes between two selected time ranges along with statistical inferences. Taxonomic behaviors like gradual increase or decrease in abundances can be easily inferred from the tabular summary generated using this workflow.

#### Workflow-5a

##### Cluster groups of taxa having similar behavior over time

An important goal while analyzing microbial time series data pertains to identification of groups of taxa which show similar trends over a time stretch. Similar temporal behavior by different bacterial taxa could arise due to reasons like symbiotic relationship between two or more bacteria. On the other hand, it is also important to know which bacterial taxa behave in temporally opposite ways, since such behavior might be an indicator of some underlying interaction or competition among them. Taxonomic groups depicting similar behavior in a selected timeframe are identified using Dynamic Time Warping (DTW) algorithm (described under “Materials and Methods” section). The output can be visually explored using interactive tree and trend plots. Each branch of the tree corresponds to a set of taxa having similar time series trends. Users can select a branch (a group of taxa having similar temporal patterns) or an individual terminal node (taxa) from the tree and visually explore the time series trends using the assistive plot.

#### Workflow-5b

##### Explore pair-wise relationship among taxonomic groups

Visualization of correlation and other similarity indices between the resident taxonomic groups often helps to gather meaningful insights. This workflow allows users to select Pearson correlation or modified DTW (referred to as TIME-DTW) index and use it to generate heatmaps. Such heatmaps are useful for visual pattern mining and the corresponding distance metric can be exported for further advanced network analysis.

#### Workflow-6

##### Explore inter taxa interactions using causality network

The existence of a strong correlation in the abundance of two or more taxonomic groups across a time scale may not always be ascertained to causation. A recent study has utilized ‘module networks’ to understand causality relationships among bacteria (Lu et al., 2017). A causation event can be ascertained between two taxonomic groups when the past values of one taxon are observed to have some information about the future values of the other. This analysis is performed in ‘TIME’ using a Granger causality algorithm (described in details under “Materials and Methods” section). The global community behavior over the whole sampled timeline is captured using interactive causality networks and trend plots. Each node in the network can be queried for its causality using interactive operations. While right clicking on a node (corresponding to a taxon) highlights the nodes (or taxa) that are affected (‘Granger caused’) by it, left clicking on the same highlights the nodes (or taxa) responsible for affecting (‘Granger causing’) its temporal changes.

#### Workflow-7

##### Cluster time points based on similar community patterns

Many microbial time series datasets are often observed to have a typical composition of constituent entities which gives rise to seasonality or periodicity of microbial communities. These similarities and differences in the proportion of the constituent taxonomic groups give rise to ‘community states’ in the microbiome. Such ‘community states’ could be useful for obtaining insights into the microbial dynamics (Gajer et al., 2012). The interactive hybrid trend plot and heatmap generated using this workflow is useful for visualizing the temporal changes in the community structure.

### Case Studies on Publicly Available Time Series Microbiome Data

We demonstrate the applicability and utility of each workflow using three publicly available time series microbiome datasets. ‘Caporaso-Dataset’ corresponds to a longitudinal metagenomic time series data of gut microbiome samples from an American healthy male and female subject, collected at regular intervals spanning a long time period (Caporaso et al., 2011). A second time series metagenomic dataset (‘Dethlefsen-Dataset’) corresponds to a study evaluating the effects two doses of antibiotic treatments on the gut microbiome of three adult American females (Dethlefsen and Relman, 2011). The third dataset (‘Gajer-Dataset’) corresponds to a temporal sampling of vaginal microbiome of 32 reproductive age women over a period of 16-weeks (Gajer et al., 2012). All the above datasets are pre-loaded into the ‘TIME’ application for users’ convenience.

#### Case Study 1: Analysis of Microbial Perturbation from Microbiome Time Series Data

**FIGURE 1:**
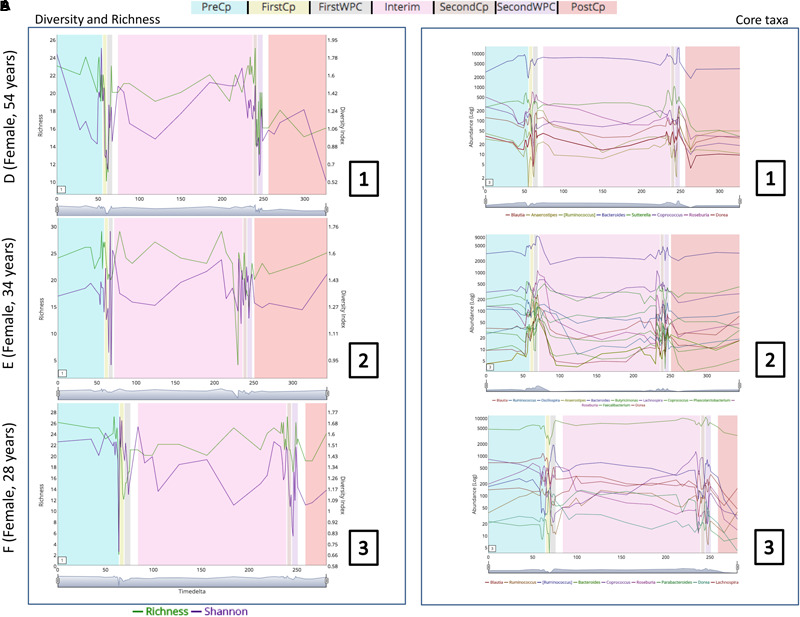
**(A)** Changes in richness and diversity of the microbial genera across the three subjects (D, E, and F of ‘Dethlefsen-Dataset’ used in the case study) especially at the time points pertaining to antibiotic treatment (‘FirstCp’ and ‘SecondCp’). **(B)** Trend plots of the core microbial genera in the three subjects show individual specific variations.

**FIGURE 2:**
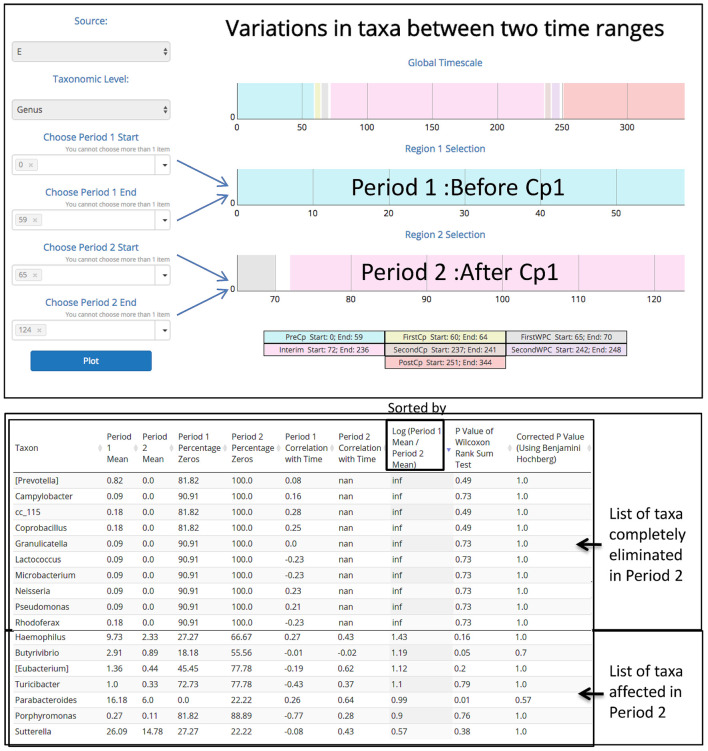
Demonstrating the utility of ‘Workflow-4’ in identifying the taxonomic groups completely eliminated and the ones mostly affected by antibiotic treatment during the first dosage period (‘FirstCp’ of ‘Dethlefsen-Dataset’ used in the case study). Two time periods (‘BeforeCp’ and ‘AfterCp’) were chosen based on the metadata.

**FIGURE 3:**
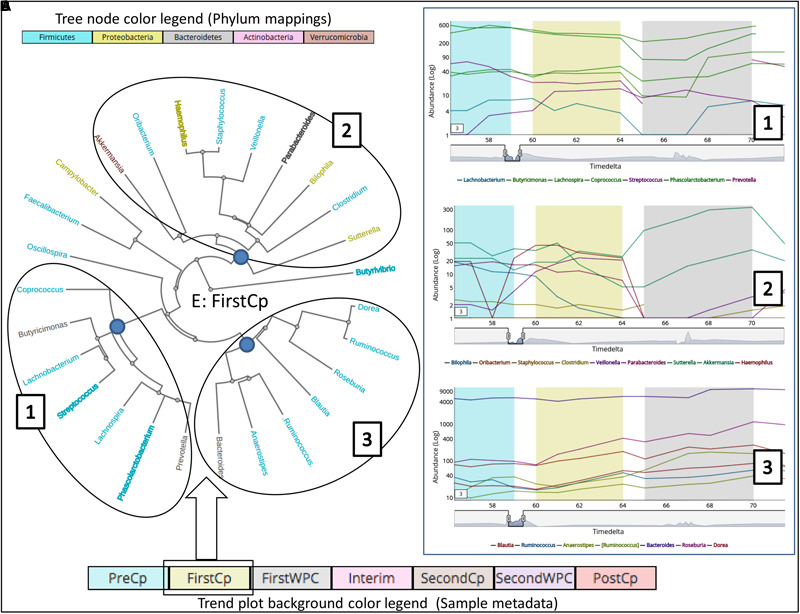
Clustering of taxonomic groups based on their temporal trends during the antibiotic treatment (‘FirstCp’ of ‘Dethlefsen-Dataset’ used in the case study) on Subject ‘E.’ The **(A)** shows a tree (radial layout) with three clusters generated using DTW-distance metric in ‘Workflow-5a.’ The **(B)** shows the corresponding trend plots for the three clusters obtained by clicking on the root node of each cluster. The genera color labels (in the tree) correspond to their respective phyla as shown in the legend while bold labels indicate non-stationary taxa.

In order to demonstrate the applicability of ‘TIME’ in analysis of perturbation, ‘Dethlefsen-Dataset’ (antibiotic treatment) was selected and various relevant workflows were used for analysis. The ‘Dethlefsen-Dataset’ had an associated metadata mapping for the time points corresponding to the different states (for all three individuals – D, E, and F), namely before antibiotic treatment (‘PreCp’), during the two doses (‘FirstCp’ and ‘SecondCp’), the week immediate post the two treatments (‘FirstWPC’ and ‘SecondWPC’), gap between the doses (‘Interim’) and the time points post treatment (‘PostCp’). A drastic drop in diversity and richness specifically at the points of perturbation (‘FirstWPC’ and ‘SecondWPC’), could be visually inferred using the diversity plots generated using TIME (**Figure 1A**). The Figure also shows the slow but incomplete recovery in diversity post perturbation, which is in line with the reported findings (Dethlefsen and Relman, 2011). The core taxa identified using ‘Workflow-1’ (at ‘genus’ level) also indicates an inter-individual variation among the three subjects (**Figure 1B**), with only four genera to be consistently common across all (*Bacteroides, Coprococcus, Roseburia*, and *Dorea*). In order to identify the taxa which are most affected by the antibiotic treatment on ‘Sample E’ (as a representative example), the Workflow-4 was employed after selecting two time stretches, namely, ‘before Cp1’ (Period 1 ranging from time point 0–59) and ‘after Cp1’ (Period 2 ranging from time point 65–124). This analysis identified the affected genera sorted by the log fold change in the mean abundances between period 1 and period 2. *Haemophilus, Butyrivibrio, Eubacterium, Turicibacter*, and *Parabacteroides* were identified to be the top five affected genera upon antibiotic treatment based of log-fold abundance (**Figure 2**) but none of them were found to be statistically significant (when evaluated with Wilcoxon Rank-Sum Test using *P*-values corrected for multiple testing). Subsequently, to gather a deeper insight into the pattern of the affected genera during perturbation or genera similarly affected during perturbation, Workflow-5a was used (selecting sample ‘E,’ time point as ‘FirstCp’ and a rare taxa cutoff of 0.5) to generate the DTW tree (**Figure 3A**). Visual inference of the tree revealed three clear clusters (**Figure 3A**), each of which were used to generate their corresponding trend plots (**Figure 3B**). While Cluster 2 seemed to contain genera whose abundance is most strongly decreased by antibiotic treatment, Cluster 1 contained the moderately affected ones. On the other hand, Cluster 3 consisted of genera which increased post perturbation, possibly due to the reduced abundances of taxa belonging to Clusters 1 and 2.

#### Case Study 2: Insights into Microbial Inter-Dependencies Using Causality Networks

**FIGURE 4:**
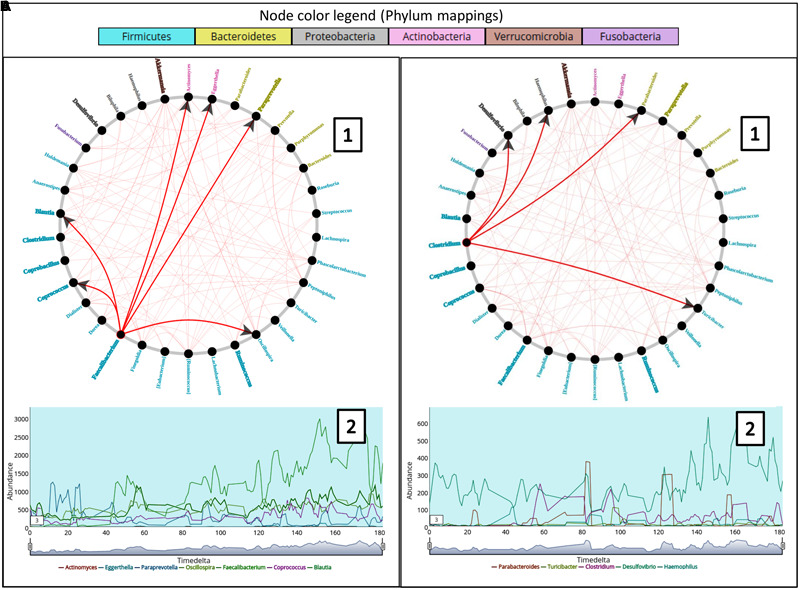
**(A,B)** Represent two composite plots for Granger causality graphs (A1 and B1) and Trend plots (A2 and B2) corresponding to genera *Faecalibacterium* and *Clostridium* respectively. Granger causality graph (A1 and B1) for the constituent taxa in the female subject of ‘Caporaso-Dataset’ used in the case study generated using ‘Workflow-6.’ The trend plots (A2 and B2) for two genera namely *Faecalibacterium* and *Clostridium* along with the genera caused (or affected) by them are displayed below the corresponding circular graphs. The arrows in the graph represent the causality relationships between the source and target nodes. The genera color labels correspond to their respective phyla as shown in the legend while bold labels indicate non-stationary taxa.

To analyze the effect of stationary genera and its relation to causality, the female subject (at genus level) from ‘Caporaso-Dataset’ (the 6 months spanning time series sampling) was used to generate the causality network using Workflow-6 (keeping a rare taxa cutoff of 0.5). The non-stationary genera information was overlaid on the network using one of the features in TIME which highlights the corresponding names (**Figure 4**). While a majority of the genera were seen to be stationary, a few exhibited non-stationary behaviors, an observation similar to an earlier study on a different gut microbiome dataset (David et al., 2014). Further, the majority of the non-stationary genera belonged to the phylum *Firmicutes*, strengthening the hypothesis of phylum level (genetically similar) resource competition (David et al., 2014). However, owing to the complexity of the community interactions in a gut microbiome, further experimental validations are required to support this hypothesis. In order to infer the effect of a non-stationary genus on others, we chose two non-stationary genera nodes (*Faecalibacterium* and *Clostridium*) from the causality network. While *Faecalibacterium* is a well documented commensal gut bacterium, a number of species belonging to *Clostridium* are known to have several pathogenic effects on human. Right clicking on these nodes enables one to highlight the edges connecting the genera affected (‘Granger caused’) by them and correspondingly displays the trend plot of all the associated taxonomic groups. A quick look into the edge connections showed that most of the genera affected by *Faecalibacterium* are non-stationary as compared to the ones affected by *Clostridium*. This observation suggests a differential influence of one taxon over others.

#### Case Study 3: Importance of Time Series Community Analysis

**FIGURE 5:**
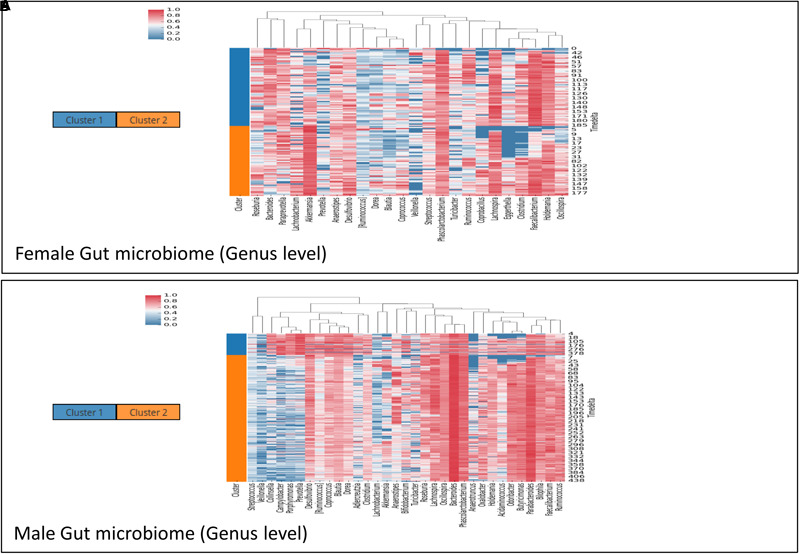
Demonstrating the utility of ‘Workflow-7’ in gathering insights on community patterns in the female **(A)** and male **(B)** subject of ‘Caporaso-Dataset’ used in the case study. The heatmaps are clustered vertically based on taxa abundance and horizontally arranged according to the two ‘community states’ (represented as ‘Cluster 1’ and ‘Cluster 2’) identified by TIME.

**FIGURE 6:**
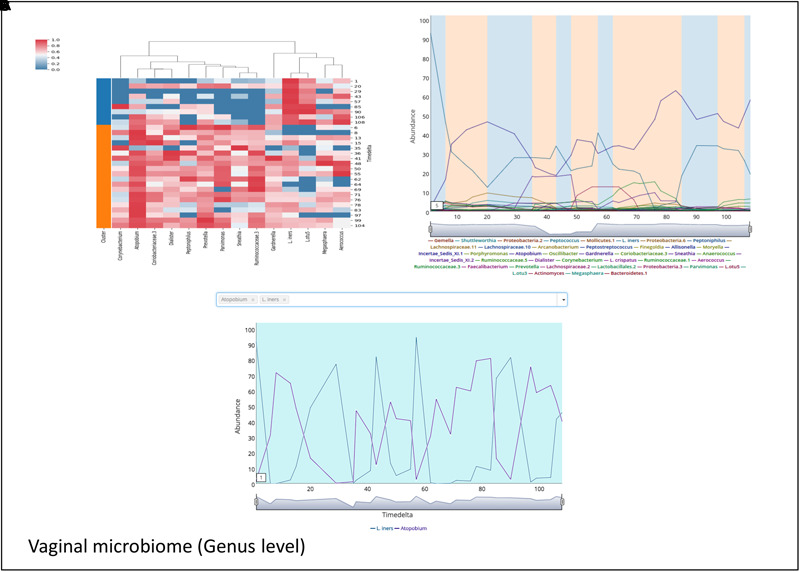
Demonstrating the periodic microbial community patterns in the vaginal microbiome of ‘Gajer-Dataset’ used in the case study. **(A)** Heatmap clustered vertically based on taxa abundance and horizontally arranged based on the two community clusters (represented as ‘Cluster 1’ and ‘Cluster 2’) identified by TIME. **(B)** Trend plots of the constituent taxa with the plot background highlighted corresponding to the ‘community state’ affiliation (in ‘blue’ and ‘orange’) of the respective time points. **(C)** Demonstrating the antagonistic behavior between the two genera *Lactobacillus iners* and *Atopobium* in the vaginal microbiome dataset used in the case study generated using ‘Workflow-2.’

Microbial communities in different body sites have been reported to exhibit differences in their compositions. These compositions are also known to change over time. For example, studies on temporal variation of human gut microbiome have reported the presence of periodic as well as non-periodic diversity patterns (Caporaso et al., 2011). Such similar temporal patterns arise due to a comparable microbial community composition across these time points. Workflow-7 of ‘TIME’ is dedicated to identify such community clusters and visualize their variations across the timeline. ‘Caporaso-Dataset’ and ‘Gajer-Dataset’ (corresponding to gut and vaginal time series microbiome, respectively) were used as a part of this analysis pipeline (results are summarized in **Figures 5, 6**, respectively). In order to consider the effect of only the ‘non-rare taxa’ (taxa which occur in at least 70% of samples), a rare taxa cutoff of 0.7 was selected and a bi-directional clustering was done (for time points and taxa). Each of the two ‘time-point clusters’ (**Figure 5**) represent a group of time points having similar microbial distributions, called ‘community states’ (see section “Materials and Methods” for details). A comparison of the female and male gut microbiome time series (‘Caporaso-Dataset’) using the above workflow revealed a clear bias of one of the two ‘community states’ in the male (**Figure 5B**) while almost an equal distribution of the two ‘community states’ was found in the female (**Figure 5A**). In male, while the dominant cluster had mainly the genera *Bacteroides* and *Parabacteroides* as distinguishable marker, other genera namely *Prevotella* and *Campylobacter* were observed to be the prominent contributors of the less dominant cluster (**Figure 5B**). On the other hand, the female microbiome had one cluster prominently dominated by *Akkermansia*, with no single clearly dominant member in the other (**Figure 5A**). To explore community states in a different body site, vaginal microbiome from subject-1 of ‘Gajer-Dataset’ was considered and analyzed using Workflow-7. A clear periodic pattern in the ‘community states’ was observed (**Figure 6B**), probably due to the prominent changes in the menstrual cycle and related hormonal changes in reproductive age females. While one ‘community state’ showed a dominance of *Lactobacillus iners*, the other showed a dominance of *Atopobium*. The genera *Atopobium* is known to be associated with bacterial vaginosis, while lactic acid producing bacteria (like *L. iners*) are known to prevent pathogen colonization by creating an acidic environment (Gajer et al., 2012). The generated heatmap (**Figure 6A**) as well as trend comparison plot using Workflow-2 (**Figure 6C**) indicate an antagonistic behavior between the above two taxa.

## Discussion

The various workflows in ‘TIME’ allow visualizing time series microbiome data as well as analyzing them to obtain meaningful biological insights. It is to be noted that a few key points need to be considered before interpreting the generated outputs and building hypotheses based on such datasets (Weiss et al., 2017). For instance, the microbial abundance files used as input for analysis represent the count of clustered sequences (OTUs) across several time points corresponding to one or more sources (samples). Regardless of strict experimental designs, not all sources as well as time points are sampled/sequenced at similar depths due to sampling constraints as well as sequencing limitations. Hence, samples sequenced at lower depth may display biased diversity estimates and consequently affect the downstream analyses. For example, workflow 4 in TIME can predict differential abundant taxa between two time stretches with increased confidence if the sequencing depths are sufficiently high and even since samples with higher number of sequence will have better estimates of abundances. Similarly, if some time points are sampled deeper than the others, it makes interpretation of transient and rare taxa difficult (workflows 1 and 3) without normalization. In addition, presence of sparse OTUs represents uncertainty in counts owing to limitations in the sequencing detection ability (since they are below the detection threshold). A majority of microbiome studies consider either a relative normalization route (OTU counts scaled to proportions) or a rarefaction based normalization step (each sample is sub-sampled to an even depth), both of which are implemented in TIME for convenience. Use of rarefaction curves can provide guidance on choosing a suitable rarefaction depth for normalization and lower the false discovery rates (Weiss et al., 2017). However, it should be kept in mind that rarefying a data might impact a number of downstream analysis workflows due to removal of a subset of the data. Moreover, time series data involving perturbation events, if normalized using rarefaction, might subdue the effect of the perturbation itself. Relative normalization on the other hand, is also prone to create several artifacts (Stämmler et al., 2016). Both rarefied as well as relatively normalized data are compositional, therefore, fluctuations in abundance of one taxon might lead to spurious fluctuations in abundance of other taxa resulting in false correlations (Weiss et al., 2017). A lack of knowledge of absolute abundance can thus impact the interpretation of the results of the analyses. For example in workflow 3, although a taxon might change in abundance and appear to be non-stationary, it may actually be not changing but taxa around it may be changing in relative abundance. Moreover, relative abundance based approaches ignore the possibility that the altered abundance itself could be a key identifier of a disease state (Vandeputte et al., 2017). It may also be noted that both relative and absolute abundances are required for obtaining a comprehensive understanding of time series microbiome data (Props et al., 2017). Additionally, data obtained from appropriately designed experiments (e.g., using replicates for each time point) will increase confidence on the obtained results. Advanced experimental protocols have also been reported (Stämmler et al., 2016) which helps in normalizing the biases arising due to differential microbial loads across samples.

The incorporated Granger causality based interaction networks in ‘TIME’ provides a way to capture the overall global microbial community behavior and is ideal for datasets having evenly sampled time-points. Variations of Granger causality have been applied earlier to decipher ecological relationships (Detto et al., 2012) and in gene expression networks (Yang et al., 2017) with reasonable success. However, not all Granger causal interactions correctly predict biological causality and are merely statistical predictions. It should also be noted that such predictions do not provide explanations regarding the origin of the interactions and could be due to an indirect influence. For instance, one time series may be a strong predictor of another time series because both are shaped together by a common underlying cause. Hence, like any other statistical prediction, a cautious interpretation of each predicted interaction is required to be made before building any hypothesis. Incorporation of functional data like metabolic co-dependencies (Levy et al., 2015) might help to strengthen the basis of a predicted interaction.

## Conclusion

The various workflows implemented in ‘TIME’ can help end users not only to perform a number of analyses, but also gain meaningful insights from the interactive visualizations. Analysis on a few well known publicly available datasets illustrate the utility of the options available in ‘TIME.’ For example, apart from obtaining information regarding the temporal effect of antibiotic treatment on human gut microbiome, ‘TIME’ could identify similarly perturbed groups of microbial genera. Additionally, the inter-microbial competition among the pathogens and commensals could also be inferred from the causality networks and stationarity analysis. In another example, the periodic changes in community structure of the vaginal microbiome were illustrated using the ‘community state’ analysis workflow. Although the scope of the case studies presented here is limited in this communication, the workflows can be further utilized to gain additional insights. We expect ‘TIME’ to be a valuable contribution in the field of microbial time series data analysis and visualization.

## Materials and Methods

‘TIME’ web application uses Python and JavaScript to execute the backend algorithms and for browser based data processing, respectively. We used the DyGraphs^1^ (DyGraphs Java Script, 2017) module for rendering time series line charts since it has the ability to handle large datasets seamlessly. Other visualizations are implemented using D3.js library (Bostock, 2017) with extensive interactive operations.

### Input Format

User data (consisting of the microbial abundance table) along with the available metadata information can be incorporated in ‘TIME’ using a simple form. The abundance table can either be provided as a standard ‘QIIME’ output (Caporaso et al., 2010) or as a tab delimited file. The metadata file is required to have information related to the source of the microbiome sample, sample names (identical to the ones in the abundance file), time stamp information along with the sample condition for each time point. A detailed description of the input files is provided in the user manual (available in the website).

### Normalization, Visual Exploration and Segregation of Microbiome Time Series Data

The microbial abundances obtained for analysis represents the count of clustered sequences belonging to the constituent taxa as operational taxonomic units (OTUs). The abundances of each OTU across different time points constitute the OTU abundance matrix. Restraints in sampling at multiple time points as well as sequencing errors result in unequal sequencing depths. ‘TIME’ provides methods to circumvent this limitation using either a proportion based or rarefaction based normalization. Rarefaction plots serve as one of the means to identify unequally sampled data points and subsequently can be used to normalize the OTU matrices such that all time points have similar counts. Users can generate a rarefaction curve for each metagenomic source by selecting either all the time points or a set of equidistant 5 or 10 time points. The generated curve can be used as a guide to select a suitable rarefaction normalization depth. Alternatively, users may proceed with absolute count data (without any normalization) or perform relative proportion based normalization. It is advisable to choose appropriate normalization method (refer to the “Discussion” section for more details).

The visual examination of the temporal trends is an important step in any time series analysis. In ‘TIME,’ all the taxa abundances at any particular taxonomic level can be viewed together as interactive line plots across the sampled timeline. An important challenge for carrying out such comparative microbial data analysis pertains to the problem of taxa abundances with different orders of magnitude (with some taxa having very high abundances and some having extremely low counts). In other words, it is difficult to visualize the trends of the lower abundant taxa owing to the dominant influence of the very high abundant ones on the plot. ‘TIME’ provides two ways to tackle this problem. While one uses ‘quartile segregation,’ the other utilizes ‘log scaling.’ In quartile segregation, the different taxa are grouped into four quartiles based on their abundance information which can be viewed separately. The very high abundant and the very low abundant taxonomic groups (or potential outliers) tend to occupy the top and bottom quartiles, respectively. The remaining quartile accommodates the taxonomic groups having the intermediate abundances. This makes sure that during visual exploration the temporal trends of the low abundance taxa do not get compressed (or dominated) by the trends of the very high abundant taxa. TIME also offers the option of log scaling the abundance values so that the trends of low abundant and that of high abundant taxa can be compared on the same plot. Additionally, the tool provides an option to view the core, persistent and the transient groups of bacteria which are reported to have distinct roles in microbial ecosystems (Caporaso et al., 2011). A taxon is considered to be persistent if it is observed in more than 20% of the time points, with at least 90% of these observations being consecutive. On the other hand, the transient taxa are those which are observed in at least 60% of the time points, with at most 75% of these observations being consecutive. However, TIME provides an option to modify these parameters (prevalence threshold and consecutive observations) for definition of core, persistent and transient in ‘workflow 1’ to accommodate differences in wide number of datasets. In addition to the above measures, the richness and diversity of the studied microbial communities are also calculated using well known indices. While richness of a microbiome denotes the unique number of constituent taxa present in each sample (at a time point), the diversity provides a measure of how evenly the taxonomic entities are distributed. Although diversity of a microbiome can be calculated using a number of ways, the widely accepted Shannon index for diversity (Shannon, 1948) has been implemented in ‘TIME.’

$$Shannon\;\;Index\;\;=-\sum_{i=1}^{R}p_{i}\;\;\text{ln}\;\;p_{i}$$

Where, *p*_i_ refers to the proportion of the abundance of the *i*^th^ taxon in the population consisting of ‘*R*’ taxa.

With implementation of each of the above methods and their corresponding visualizations, an interactive operation for selecting a ‘subplot window’ of the plotted timescale is presented. This feature enables users to graphically choose the start and end time points using simple mouse operations and visualize the selected time range. The ‘subplot window’ can then be dragged along the time-scale with a zoomed view. The moving average of a time series can also be specified using a text box available at the bottom corner of the plot window. This feature smoothes the short term fluctuations in the time series data and shows the overall trends (and cycles) across a longer timescale.

### Identification of Stationary Taxonomic Groups

Stationarity of taxa in microbial time series data is important to understand inter-microbial competition (David et al., 2014). A taxon is considered stationary if its mean, variance, covariance, and autocorrelation are constant over time, due to the absence of a unit root process. A unit root process is said to be present in a time series if its autoregressive model has an estimated coefficient close to one. The presence of a unit root indicates that a perturbation in the value of the entity in the time series has a persistent impact on its future values and hence a cause of non-stationarity. The most commonly used method for calculating stationarity is the Augmented Dickey Fuller (ADF) Test. While the null hypothesis of the ADF Test is that there is a unit root process governing the dynamics of the entity (taxon), the alternate hypothesis states that there is no unit root. The ADF test statistic is a number, the more negative it is, the stronger will be the confidence with which the null hypothesis can be rejected. ‘TIME’ allows identification of microbial groups detected to be stationary and non-stationary and lists the same in a searchable table with options to export the results. The stationarity information corresponding to each taxon is also used to augment other plots in the tool along with the phylogeny information.

### Generation of Inter-Microbial Causality Network

One of the important objectives in time series studies pertains to identifying causal relationships among entities. Causality aims to find the direct interactions between entities such that one entity can trigger/suppress or be triggered/suppressed by the other. It should be noted that causation should not be confused with correlation. For example, two taxa (‘A’ and ‘B’) in a microbiome time series dataset may be correlated, but may not have any causal relationships. Granger Causality (Granger, 1969) is one of the most well established statistical tests for checking causality among two time series. The basic premise of this method is that, if one variable causes another, the past values of the former must have some information about the future values of the latter (which is not available otherwise). For example in a microbiome time series data, if taxon A affects taxon B, the future values of taxon B can be better predicted using the past values of both taxa A and B, rather than using the past values of taxon B alone. In order to ascertain if taxon A influences taxon B, two regressions are performed. In one, past values of taxa A and B are used to predict the present values of taxon B. In the other, only the past values of taxon B is used to predict the present values of taxon B. If a significant increase of the goodness of fit of the former regression over the latter is observed, then taxon A is said to ‘Granger cause’ taxon B.

Since a typical microbiome time series data has more than two entities (taxa), ‘Granger Lasso Causality’ (Hlaváčková-Schindler and Pereverzyev, 2015) can be used to find causal relationships among all taxa. Thus, apart from the ‘Pairwise Granger causality’ (described above) for all possible taxa pairs, ‘Granger-Lasso’ method has also been implemented in ‘TIME.’ The LASSO (Least Absolute Shrinkage and selection operator) is one of the most well known and widely used methods for feature selection and regularization in machine learning. LASSO works by adding a regularizing penalty to the sum of squared errors. This objective function is minimized (by optimization) for estimating the values of the coefficients of regression (thus reducing the weightage/coefficients of the unimportant predictors), thereby finding the best set of predictors for every variable. Granger-Lasso utilizes the LASSO methodology for identifying causal relationships among all entities (taxa) in multivariate microbiome time series dataset (Arnold et al., 2007). Another option allowing selection of causality pairs predicted by both ‘Pairwise Granger’ and ‘Granger-Lasso’ is implemented for improved Granger Causality predictions. All these three methods are available in ‘TIME’ which can be finally used to generate a directed causality network.

### Identifying Taxonomic Groups Having Similar Temporal Patterns

In time series datasets, it is not only important to evaluate temporal changes of different entities and the causal relationships among them, but also to identify entities which exhibit similar temporal patterns. The Euclidean distance based clustering of entities is unsuitable for identifying similar temporal patterns since this distance measure does not take into account the distortion across time series (Keogh and Ratanamahatana, 2005). In other words, the temporal behavior of two taxa which are out of phase is assigned a high value by Euclidian measure. On the other hand, Dynamic Time Warping (DTW) gives due importance to the phase displacement and obtains the optimal alignment between the two time series (Berndt and Clifford, 1994). DTW uses a dynamic programming based approach to align and score the similarity of the temporal patterns corresponding to two entities (taxa in the case of microbial time series). Since the DTW algorithm is relatively slow with a worst case time complexity of O(n^2^), a modified DTW algorithm (Sakoe and Chiba, 1990) is implemented in ‘TIME’. In this algorithm, a constraint is applied in such a way that a limited number of cells are evaluated during computing the cost matrix of the alignment, thereby making the overall computation process much faster (Salvador and Chan, 2007). ‘TIME’ uses the calculated pair-wise DTW distances among the different taxa for hierarchical clustering. The resulting dendrograms can be viewed as trees in standard or radial layouts. One of the limitations of the DTW distance pertains to the inability to interpret the distance score easily as it does not fall in a definite range. Therefore, it is desirable to have a modified score with a definitive range that can be universally interpreted. In order to achieve this, we introduce a new method for calculating the distance between two time series, called the ‘TIME-DTW Distance,’ In a microbial time series data, one taxon can have a difference of several orders of magnitude with another, but their time series may have similar overall shape. Thus, a standard normalization step is first applied to minimize such differences. Following this, the DTW distance is calculated and normalized by the average ‘sum of the absolute difference’ (SAD) between each time series and its ‘mirror image’ (Supplementary Material). The resultant value (‘TIME-DTW distance’) will hence always fall between a range of zero and one. ‘TIME’ allows an easy and interactive way to explore the results using a ‘clustered heat map.’ In addition to using the TIME DTW Distance as the measure of similarity/dissimilarity, the pairwise similarity between taxa can also be viewed using Pearson Correlation coefficient. The resulting heatmaps are hierarchically clustered based on their distances along the vertical axis, and taxonomic hierarchies along the horizontal axis.

### Understanding Community Structure Based on Similarities across Time Points

Apart from understanding the temporal similarities among the resident entities (taxa), clustering of time points having similar entity distribution is expected to yield valuable insights regarding the microbial community dynamics. The identified time points having similar taxonomic distributions (i.e., phylotype proportions) can be considered as a ‘community state’ (Gajer et al., 2012). Jenson Shannon divergence (JSD) metric has been utilized earlier to identify such ‘community state’ in microbiome time series data (Gajer et al., 2012). In ‘TIME,’ a modification of the method is implemented to make it applicable for any microbiome time series. The taxa abundances are first normalized to generate probability distributions, which are then used to calculate the JSD among the different time points. Thus, a pairwise JSD matrix is obtained for all time points. Since, the *K*-medoids clustering algorithm (Jin and Han, 2011) is known to be robust to noise and outliers (as compared to *K*-means), it was utilized for clustering the time points using the generated JSD matrix. The number of clusters can be chosen by the user based on visual inspection. Along with clustering the different samples based on their microbial community structure, it is often useful and sometimes necessary to find the drivers of the cluster, i.e., the most dominant taxa among the clusters. Keeping this in view, ‘TIME’ also provides the option to view the (normalized) relative abundances of the taxa across different clusters and different time points as a heatmap, which helps in visual exploration and determination of the distinctive/driver taxa or groups of taxa.

## Author Contributions

KB, BK, and SM conceived the idea and designed the overall methodology. KB and BK implemented the algorithms and developed the web server. BK performed the case studies. BK, KB, and SM analyzed the results and drafted the manuscript. All authors read and approved the final manuscript.

## Conflict of Interest Statement

All authors were employed by the company Tata Consultancy Services Ltd.

## Footnotes

1 http://dygraphs.com/
