# Corrigendum: ‘TIME’: A Web Application for Obtaining Insights into Microbial Ecology Using Longitudinal Microbiome Data

**DOI:** 10.3389/fmicb.2020.605295

**Published:** 2020-11-11

**Authors:** Krishanu D. Baksi, Bhusan K. Kuntal, Sharmila S. Mande

**Affiliations:** ^1^Bio-Sciences R&D Division, TCS Research, Tata Consultancy Services Ltd., Pune, India; ^2^Chemical Engineering and Process Development Division, CSIR-National Chemical Laboratory (NCL), Pune, India; ^3^Academy of Scientific and Innovative Research (AcSIR), Ghaziabad, India

**Keywords:** time series, microbiome, community state, visualization, clustering, Granger causality algorithm, web server

In the published article, there was an error in affiliation for Bhusan K. Kuntal. The “Academy of Scientific and Innovative Research, CSIR-National Chemical Laboratory Campus, Pune, India” should be displayed as two different affiliations: “Chemical Engineering and Process Development Division, CSIR-National Chemical Laboratory (NCL), Pune, India” and “Academy of Scientific and Innovative Research (AcSIR), Ghaziabad, India.”

In the published article, there was an error in the Conflict of Interest Statement. It should read:

All authors were employed by the company Tata Consultancy Services Ltd.

The authors apologize for these errors and state that they do not change the scientific conclusions of the article in any way. The original article has been updated.

