# Supplementary material for: ‘TIME’: A Web Application for Obtaining Insights into Microbial Ecology Using Longitudinal Microbiome Data
Source: Front Microbiol. 2018 Jan 24;9:36. doi: 10.3389/fmicb.2018.00036 (PMC5787560; doi:10.3389/fmicb.2018.00036)
Supplement: Supplementary file 1 [file Image_1.PDF]

Supplementary Material

‘TIME’: A web application for obtaining insights into microbial ecology using longitudinal microbiome data

Krishanu Das Baksi [§1], Bhusan K. Kuntal[§1,2] and Sharmila S. Mande[*1]

[1] Bio-Sciences R&D Division, TCS Research, Tata Consultancy Services Ltd., 54-B Hadapsar Industrial Estate, Pune 411 013, India.

[2] Academy of Scientific and Innovative Research (AcSIR), CSIR-National Chemical Laboratory Campus, Pune 411 008, India

§ Equal contribution * Corresponding Author (sharmila.mande@tcs.com)

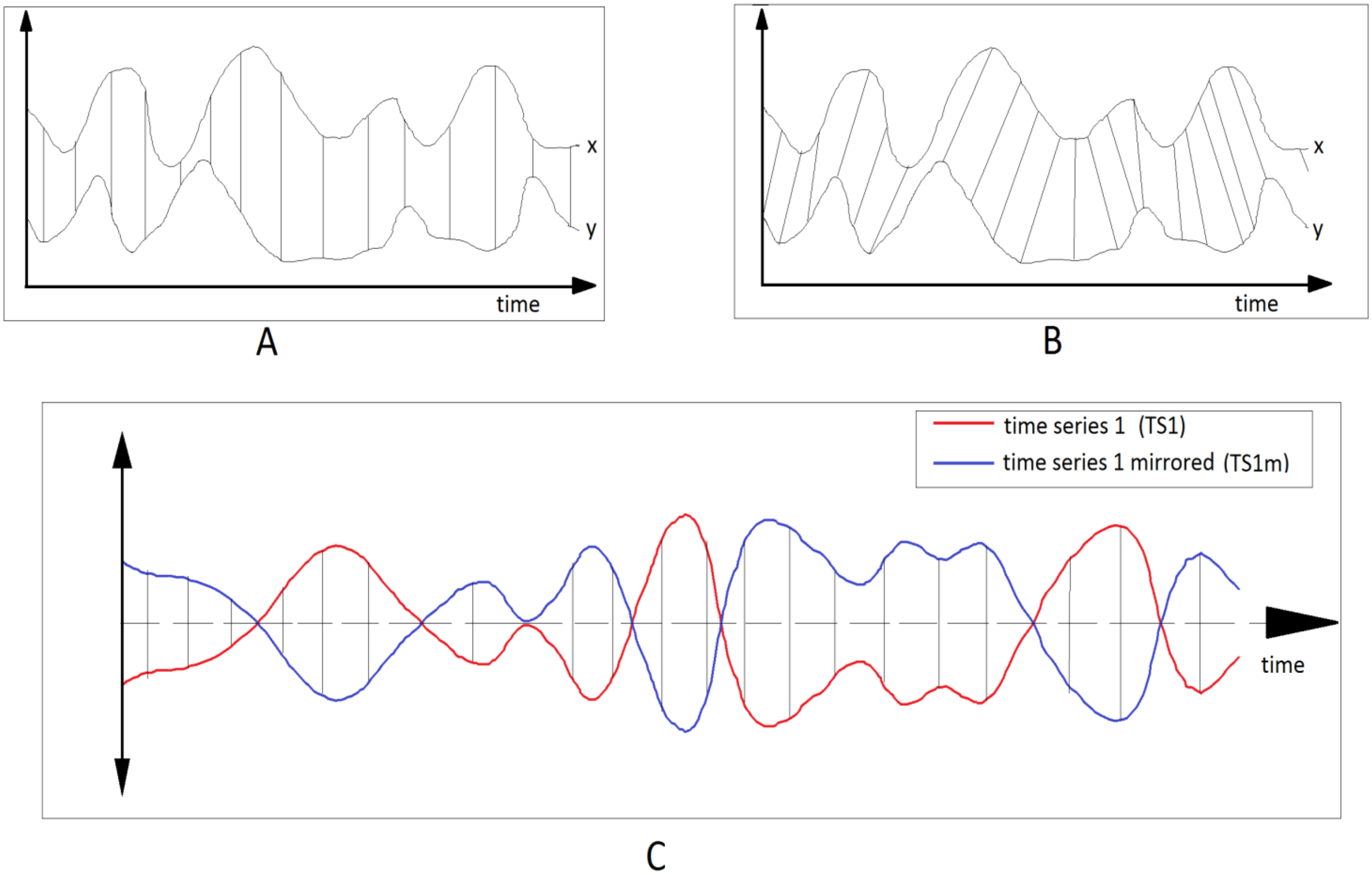

Figure A: The Distance between the two time series (x and y) is calculated as the Sum of the Absolute Differences (SAD) between them.

Figure B: The Distance (between x and y) is calculated by the Dynamic Time Warping(DTW) algorithm with some constraints. If however the constraint is set to zero (i.e. warping is not allowed), the DTW Distance will approach its maximum value (for the corresponding pair of time series) and also will be equal in magnitude to the SAD. Since the DTW Distance tries to find the minimum distance between two time series, it will always be less than or equal to the SAD.

Figure C: The two plots TS1(red) and TS1m (blue) represent standardised two time series (i.e. time series with mean = 0 and standard deviation = 1). Note that these are just mirror images (vertically flipped image) of each other. The SAD between a standardised time series and its mirror image will be maximum (MaxSAD), i.e. no other time series (with zero mean and unit standard deviation) can have SAD (with the original time series) greater than the MaxSAD.

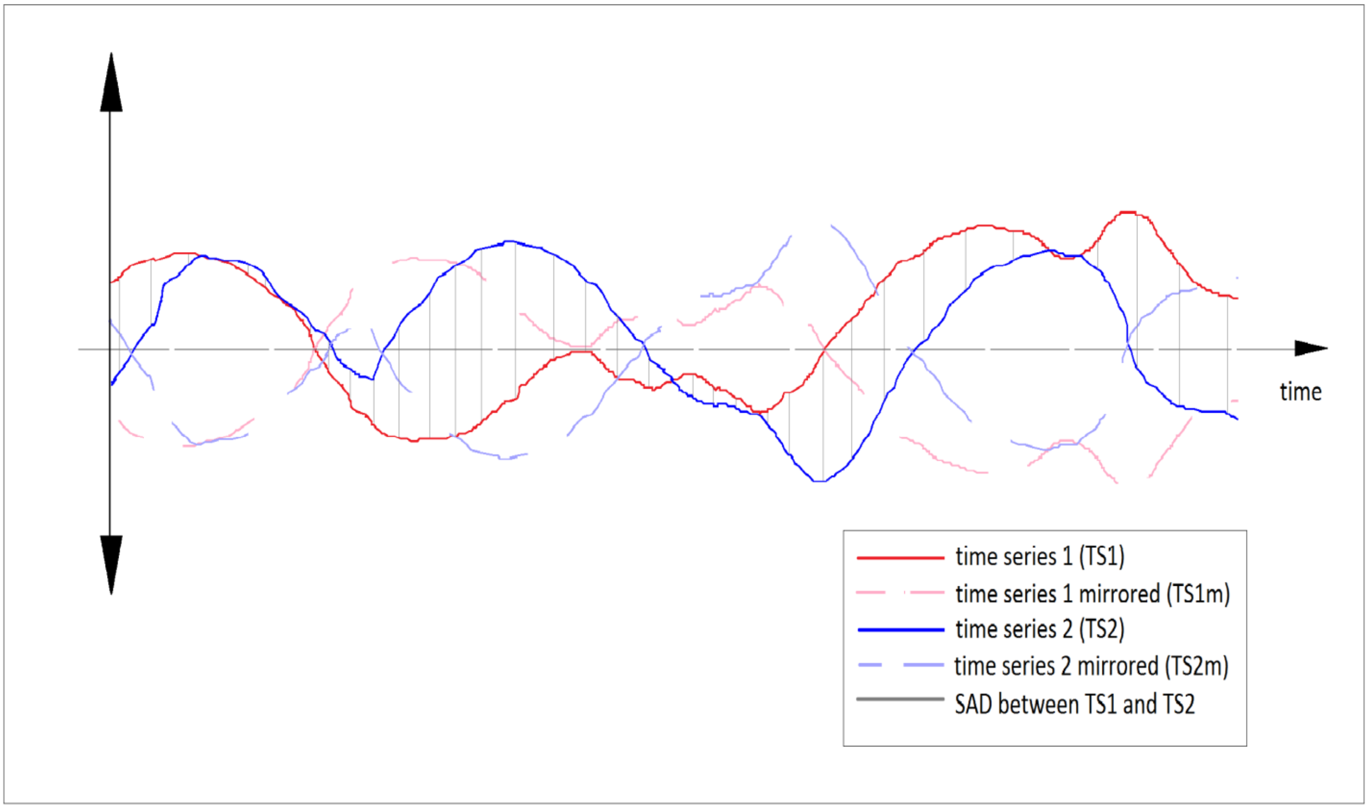

D

Figure D: It can be easily shown that for two arbitrary standardised time series(suppose TS1 and TS2), the SAD between them will always be less than or equal to the average of $MaxSAD_{TS1}$ and $MaxSAD_{TS2}$ ($AvgMaxSAD_{TS1, TS2}$ = $(MaxSAD_{TS1} + MaxSAD_{TS2}) / 2$)). SAD between TS1 and TS2 ($SAD_{TS1, TS2}$) will be equal to AvgMaxSAD if and only if the two time series are mirror images of each other, in which case the ratio of $SAD_{TS1, TS2}$ and AvgMaxSAD will equal one. If on the other hand, TS1 and TS2 are exactly identical, the $SAD_{TS1, TS2}$ will be zero, thereby making the ratio of $SAD_{TS1, TS2}$ and AvgMax $SAD_{TS1,TS2}$ zero as well.

Therefore, the ratio of the SAD and AvgMaxSAD between two time series will always lie between zero and one. Now since SAD is the maximum value that the DTW Distance can take, if the ratio of

the DTW Distance and the AvgMaxSAD between two time series is taken, it should also lie in the range of zero to one. This ratio is the TIME DTW Distance.

$$\text{TIME DTW Distance} = DTW(TS1, TS2) / ((MaxSAD_{TS1} + MaxSAD_{TS2}) / 2)$$
